# Venous thromboembolism 2011–2018 in Stockholm: a demographic study

**DOI:** 10.1007/s11239-019-01966-y

**Published:** 2019-10-01

**Authors:** Per Wändell, Tomas Forslund, Helene Danin Mankowitz, Anna Ugarph-Morawski, Staffan Eliasson, Frieder Braunschwieg, Margareta Holmström

**Affiliations:** 1 Division of Family Medicine and Primary Care, Department of Neurobiology, Care Sciences and Society, Karolinska Institutet, Huddinge, Sweden; 2 Department of Medicine, Centre for Pharmacoepidemiology, Karolinska Institutet, Solna, Sweden; 3 Public Healthcare Services Committee, Department of Healthcare Development, Region Stockholm, Stockholm, Sweden; 4 Academic Primary Care Centre, Region Stockholm, Stockholm, Sweden; 5 Department of Medicine, Karolinska Institutet, Solna, Sweden; 6 Themes Heart and Vascular, Karolinska University Hospital, Solna, Sweden; 7 Team Coagulation Disorders, Karolinska University Hospital, Solna, Sweden; 8 Division of Family Medicine and Primary Care, NVS Department, Karolinska Institutet, Alfred Nobels Allé 23 (D2), 141 83 Huddinge, Sweden

**Keywords:** Deep venous thrombosis, Pulmonary embolism, Gender, Epidemiology, Trends, Anticoagulant agents

## Abstract

Venous thromboembolism (VTE) is an important cause of morbidity and mortality in Western countries. The incidence rate of VTE is estimated at 1–2 cases per 1000 annually. This study was a population-based cohort study of previously treatment naïve patients with a first occurrence of venous thromboembolism (VTE), using data from the administrative health data register of the Stockholm Region 2011–2018. Data on anticoagulant treatment was taken from the Swedish Prescribed Drug Register. We also analyzed all VTE events between 2011 and 2018. Altogether 14,849 naïve incident VTE cases were identified. In 2011 the majority of patients with a first episode of VTE were prescribed warfarin versus non-vitamin K antagonist oral anticoagulants (NOACs), 1144 versus 5. In contrast in 2018, the majority of patients were treated with NOACs, 1049 versus 59 treated with warfarin. Treatment with low molecular weight heparin only decreased from 814 to 683 patients. The frequency of all VTE events in the population increased over time from 1.88/1000 to 1.93/1000 (p = 0.072), and PE diagnoses increased from 0.69/1000 to 0.76/1000 (p = 0.003). In conclusion, during 2011–2018 there has been a shift of prescription of warfarin to a clear predominance of NOACs in the treatment of VTE in the Stockholm Region, in line with current recommendations. In the clinical situation, treatment has been simplified as monitoring of warfarin has decreased substantially. PE events increased during the time period in the population even if the increase was rather modest, while all VTE events did not increase significantly.

**Electronic supplementary material:**

The online version of this article (10.1007/s11239-019-01966-y) contains supplementary material, which is available to authorized users.

## Highlights

- Venous thromboembolism (VTE) is an important cause of morbidity and mortality
- Population-based cohort study from Stockholm Region of patients with VTE 2011-2018
- Anticoagulant treatment has shifted from warfarin to NOACs during the period
- Pulmonary embolism has increased during the period
- Further studies especially on the association to cancer are warranted

## Introduction

Venous thromboembolism (VTE) is an important cause of morbidity and mortality in Western countries, with an incidence rate of 1–2 cases per 1000 and year [1–7]. In Sweden, the incidence of deep venous thrombosis (DVT) is estimated at 1.67 per 1000 per year, with pulmonary embolism (PE) 0.8 per 1000 per year [8].

Both acquired and genetic risk factors contribute to the risk of VTE [9, 10]. A large proportion of VTE develop after previous hospitalizations and surgical procedures [11]. Important acquired risk factors for VTE include [9–11]: age, major surgery, trauma, immobilization, malignancy, prior VTE, presence of central venous lines, chronic heart failure, estrogen therapy, pregnancy and the post-partum period. Age is a major risk factor for VTE independent of gender [2]. A recently published large study with 1.1 million participants from 76 different cohorts, found that older age, smoking, and adiposity were consistently associated with higher VTE risk [12].

There have been different and somewhat contradictory results in studies regarding incidence of VTE, with a review concluding, that “the occurrence of VTE seems to be relatively constant, or even increasing” [7]. One study from the US showed an increased incidence [6], while a French study found a decreased incidence [3]. The latter study found different trends as regards DVT and PE, with a decreased incidence of DVT but not of PE.

The aim of the present study was to explore the anticoagulant treatment and incidence of VTE in Region Stockholm especially regarding time trends.

## Method

This study was a population-based cohort study of previously treatment naïve patients with a first occurrence of venous thromboembolism (VTE; for ICD-10 codes see Supplementary Table 1) using data from the administrative health data register of Region Stockholm (Vårdanalysdatabasen, VAL), i.e., the regional healthcare data warehouse [13–16]. It contains anonymized data regarding diagnoses, age, sex, prescription claims, hospitalizations and other healthcare consultations, migration and death for all individuals in the region (2.3 million inhabitants). Data regarding diagnoses from primary care is available since 2003, and for secondary care (outpatient visits and hospitalization) since 1993. VAL also contains individual level data on all prescription drugs dispensed anywhere in Sweden to inhabitants in the region since July 2010: amounts, expenditures and reimbursement, the age and gender of the patient, co-payments and prescriber category. This information is derived from the Swedish Prescribed Drug Register (National Board of Health and Welfare) [17, 18]. The study was approved by the Regional Ethical Review Board in Stockholm (EPN Dnr: 4-1/2018).

The index date of individuals with a first occurrence of VTE (See Supplementary Table 1) in secondary care from 2011 to 2018 was identified. Patients with an index date during the period January 1st 2011 until December 31st 2018 and a first claim of either an oral anticoagulant (OAC) or low-molecular weight heparin (LMWH) within ± 30 days of the index date were included. The wash-out period to define a new introduction of an OAC or LMWH was 6 months. The individuals were considered treated with OAC when both an OAC and LMWH were claimed. Individuals with a previous VTE diagnosis earlier than 2011 were excluded, as well as patients with VTE during pregnancy or post-partum period (ICD-10 codes O22.2, O22.3, O22.5, O22.9, and O87).

Age at index date was used for further analyses. Co-morbidity was defined as at least one registration of each diagnosis (ICD-code) as either a main or a secondary diagnosis in either primary or secondary care (outpatient visits and hospitalization) during 5 years up until the index date. Other drugs at baseline were included if they were claimed during 6 months prior up until the index date of the OAC of inclusion. See Supplementary Table 1 for definitions of co-morbidity diagnoses (with ICD-10 codes) and Supplementary Table for drug treatments (with ATC codes).

### Statistical analysis

Baseline data were registered, with means and standard deviations or numbers and frequencies for men and women. Use of anticoagulant medications were registered, with warfarin, low molecular weight heparin or NOACs, both for all treatment naïve VTE cases as well as for cases with a previous registered cancer. Type of previous cancer was also noted.

We also calculated the incidence rate of all diagnoses of VTE and PE, with or without a claim of either an oral anticoagulant (OAC) or low-molecular weight heparin (LMWH) within ± 30 days of the index date during the time period regardless of earlier treatment, using direct age standardization in the population at risk. Time trend for registered diagnoses over time were estimated by linear regression models.

## Results

In Table 1 the identified cases of VTE among men and women in Region Stockholm 2011–2018 are shown, with age profile and registered comorbidity (comorbidity ICD-10 codes in Supplementary Table 1). The mean age in this study was 69.0 years for women and in men 65.0 years and the majority of patients were older than 60 years. Few cases of VTE were diagnosed in children. Data regarding comorbidities showed high proportions; i.e. 68.0% of the patients suffered from hypertension, 24.8% of the patients had cancer, 20.2% diabetes mellitus, 18.7% stroke, 17.5% heart failure, and finally 16.8% had atrial fibrillation.

**Table 1:** New cases of venous thromboembolism (VTE) diagnoses in Stockholm county 2011–2018, with baseline data on ages and co-morbidities

	Women	Men	Total
	(n=7537)	(n=7312)	(n=14,849)
Mean age (SD)	69.0 (17.1)	65.3 (14.4)	67.2 (15.9)
VTE per age-groups			
0–19 years	29 (0.4)	30 (0.4)	59 (0.4)
20–39 years	539 (7.2)	327 (4.5)	866 (5.8)
40–59 years	1236 (16.4)	1890 (25.9)	3126 (21.1)
60–79 years	3470 (46.0)	3914 (53.5)	7384 (49.7)
80–99 years	2253 (29.9)	1148 (15.7)	3401 (22.9)
≥ 100 years	10 (0.1)	3 (0.04)	13 (0.1)
Diagnoses			
Atrial fibrillation	1232 (16.4)	1268 (17.3)	2500 (16.8)
CHF	1460 (19.4)	1138 (15.6)	2598 (17.5)
Hypertension	5209 (69.1)	4883 (66.8)	10092 (68.0)
Diabetes	1312 (17.4)	1694 (23.2)	3006 (20.2)
Vascular disease	1541 (20.5)	1650 (22.6)	3191 (21.5)
Stroke	1422 (18.9)	1357 (18.6)	2779 (18.7)
Cancer	1803 (23.9)	1880 (25.7)	3683 (24.8)
Dementia	515 (6.8)	256 (3.5)	771 (5.2)
Anaemia	1155 (15.3)	865 (11.8)	2020 (13.6)
Alcoholism	195 (2.6)	489 (6.7)	684 (4.6)
Upper GI bleeding	62 (0.8)	60 (0.8)	122 (0.8)
Intra-cranial bleeding	158 (2.1)	176 (2.4)	334 (2.3)
Any severe bleeding	571 (7.6)	488 (6.7)	1059 (7.1)
Kidney disease	467 (6.2)	562 (7.7)	1029 (6.9)
Liver disease	116 (1.5)	145 (2.0)	261 (1.8)
Obesity	636 (8.4)	415 (5.7)	1051 (7.1)
COPD	761 (10.1)	517 (7.1)	1278 (8.6)
Falls	1028 (13.6)	626 (8.6)	1654 (11.1)

The distribution of the identified newly diagnosed VTE cases by year, and anticoagulant treatment, is shown in Table 2 (ATC codes in Supplementary Table 2). Treatment shifted markedly over time, with a decreased use of warfarin and an increased use of NOACs. Patients with a previous cancer registered were more often treated by LMVH compared to all VTE cases, 65.7% versus 39.9%. Numbers and frequencies of associated cancer cases are shown in Supplementary Table 3. The highest proportions of registered cancers exceeding 10% were skin cancer (19.5%), prostate cancer (18.4%), breast cancer (14.6%), and metastatic cancers (metastatic lymph node 13.9%, metastasis in lung, thorax, liver or other gastrointestinal organs 13.8%, and other sites of metastasis 10.5%).

**Table 2:** Time trends of medications of new cases of venous thromboembolism (VTE) in previously treatment naïve patients in Stockholm County 2011–2018

	2011	2012	2013	2014	2015	2016	2017	2018	2011–2018
All VTE cases	1963	1860	1893	1834	1806	1864	1838	1791	14,849
Deep vein thrombosis	931	864	913	874	860	855	768	766	6831 (46.0%)
Pulmonary embolism	799	768	797	766	752	749	802	737	6170 (41.6%)
Other VTE	233	228	183	194	194	260	268	288	1848 (12.4%)
Treatment all VTE									
Warfarin	1144	1019	886	712	402	220	114	59	4556 (30.7%)
LMWH	814	834	834	749	698	690	625	683	5927 (39.9%)
NOAC	5	7	173	373	706	954	1099	1049	4366 (29.4%)
Treatment of VTE with previous cancer									
Warfarin	183	134	138	101	65	35	16	12	684 (18.6%)
LMWH	327	337	353	336	287	273	235	273	2421 (65.7%)
NOAC	0	1	14	53	82	111	164	153	578 (15.7%)

*LMWH* low molecular weight heparin, *NOAC* non-vitamin K oral anti-coagulants

All registered VTE diagnoses categorized according to age group during the time period are shown in Table 3. When using age-standardized data, the total number was 31,219, i.e., around three times as many as the registered new cases, with 12,027 PE diagnoses. The frequency of VTE diagnoses increased from 1.88/1000 to 1.93/1000 (p value for linear trend = 0.072), with a mean frequency of 1.90 (SD 0.021); and of PE diagnoses from 0.69/1000 to 0.76/1000 (p-value for linear trend 0.003), with a mean frequency of 0.73 (SD 0.034). When also using a claim of either an oral anticoagulant (OAC) or low-molecular weight heparin (LMWH) within ± 30 days of the index date, the frequency of VTE diagnoses remained on the same level, i.e., 1.61/1000 (p-value for linear trend 0.39), with a mean over the years of 1.60/1000 (SD 0.026); and of PE diagnoses 0.60/1000 to 0.65/1000 (p-value for linear trend 0.007), with a mean over the years of 0.63/1000 (SD 0.028).

**Table 3:** All patients with a first recorded diagnosis of VTE, and with a first recorded diagnosis of pulmonary embolism, with a diagnosis only or who also were claimed either OAC or LMWH within 30 days before or after the index date, in Region Stockholm the years 2011–2018

	2011	2012	2013	2014	2015	2016	2017	2018	2011–2018
Standardized population	2,058,458	2,058,458	2,058,458	2,058,458	2,058,458	2,058,458	2,058,458	2,058,458	2,058,458
Diagnosis only									
Age standardized cases	3880	3890	3873	3848	3885	3970	3905	3967	31,219
VTE (‰)	1.88	1.89	1.88	1.87	1.89	1.93	1.90	1.93	1.90
Age standardized cases	1427	1414	1473	1520	1482	1520	1625	1566	12,027
PE (‰)	0.69	0.69	0.72	0.74	0.72	0.74	0.79	0.76	0.73
Diagnosis and treatment									
Age standardized cases	3324	3267	3256	3220	3267	3392	3315	3319	26,361
VTE (‰)	1.61	1.59	1.58	1.56	1.59	1.65	1.61	1.61	1.60
Age standardized cases	1241	1213	1285	1300	1272	1334	1395	1338	10,378
PE (‰)	0.60	0.59	0.62	0.63	0.62	0.65	0.68	0.65	0.63

Direct age standardized incidence in the population at risk. For 2011–2018 total number of cases, and means are given

Most cases of VTE occurred in the age-group 60–79 years, 45.1%, followed by the age-group 40–59 years, 24.7%, and the age-group 80–99 years, 19.6% (data without age standardization, Supplementary Table 4). Most of the PE cases also occurred in the age-group 60–79 years, 49.5%, followed by the age-group 80–99 years, 22.7%, and the age-group 40–59 years, 20.1% (data without age standardization, Supplementary Table 5). The total population in Region Stockholm over the years 2011–2018 are given in Supplementary Table 6.

## Discussion

The main findings of this study was a clear shift in treatment of VTE during 2011–2018 from predominantly warfarin in the beginning of the time period to predominantly NOACs at the end of the time period. We also found a trend with an increasing rate of PE events during the time period 2011–2018, while not of all VTE events.

Regarding the time trend for VTE and PE diagnoses, earlier studies have observed conflicting results, with an increased incidence in a US study [6], and a decreased incidence in a French study [3]. We found an incidence of all VTE cases of around 1.9 per 1000 inhabitants, compared to 0.66 per 1000 inhabitants in the study of the city of Malmö in southern Sweden [19]. The incidence of PE cases was approximately 0.7 per 1000 inhabitants, compared to 0.2 per 1000 in the Malmö study. In an earlier study the incidence of DVT in the city of Malmö was 1.9 per 1000 inhabitants [1], i.e., higher than in the study by Isma et al. [19], and also higher than in the present study, i.e., around 1.2 cases of DVT per 1000 inhabitants. The incidence of DVT in a US study was close to that in the study by Nordström et al. i.e., 1.9 per 1000 inhabitants [20].

This register study included 14,189 cases from Region Stockholm with a first episode of VTE during 2011–2018. To our knowledge this is one of the largest studies in this field. Age distribution and distribution between sexes is in accordance with other studies. The number of children with VTE was low which also is in accordance with other published results. Cancer is a well-known risk-factor for VTE as shown by our data. Other cardiovascular co-morbidities were also common, especially hypertension.

The observed shift in anticoagulant treatment pattern, with a strongly decreased use of warfarin and an increased use of NOACs has already been shown for treatment in atrial fibrillation during the same time period [21, 22]. Recommendations for treatment with anticoagulation of VTE is available on the so called Wise List [23], which is an official recommendations which strongly affects prescription patterns in Region Stockholm [24]. The first recommendation for NOAC for treatment of VTE was official in December 2014 and influenced prescription pattern for VTE thereafter.

A review and meta-analysis of NOACs versus warfarin in patients with VTE or atrial fibrillation concluded, that the risk of major bleeding events decreased by between 32 and 69% for dabigatran, rivaroxaban, and apixaban compared with vitamin K antagonists, based on 7 RCTs [25]. Furthermore, the risk of intracranial bleedings decreased by between 61 and 86% of NOACs with the exception of dabigatran. According to a review, PE incidence rates seem to increase over time, “possibly due to increased monitoring, improved diagnosis and an aging population” [26]. Furthermore, in the same review was concluded, that NOACs seem to exert advantages over warfarin, especially in some subgroups, such as elderly, fragile patients, and also patients at high risk of recurrent VTE events, or patients with high risk of bleeding complications.

The age distribution pattern differed between all VTE cases and PE cases, with the peak in higher ages for PE cases.

This study has some limitations. This is an observational study, why causal associations could not be claimed. Data are taken from registers, without access to other clinical data, such as smoking, BMI, blood pressure or laboratory values. Misclassification is reported to be relatively common in VTE, especially in DVT [27]. In a recent publication from Sweden 2450 VTE diagnoses was validated manually [28]. Misclassification of VTE diagnosis was reported to occur in 16.4% of DVT-cases and in 1.1% of PE-cases. Thus, data on DVT should be interpreted with some caution. However, we also used treatment data, which give our result further strength [29], and we had also access to all registered VTE and PE diagnoses during the time period.

Our study also has strengths. It is a large study with more than 14,000 cases included in the main study. Overall the Swedish registers are known to be of high quality, including the Swedish Prescribed Drug Register [17, 18]. The VAL register in Region Stockholm includes diagnoses from both hospital care, primary care and other open specialist care, and has been used in several other studies, including atrial fibrillation [15], and studies of other diagnoses [30]. Furthermore, Region Stockholm has a population of 2.3 million inhabitants.

In the clinical situation, treatment has been simplified as monitoring of warfarin has decreased substantially. Besides, treatment with anticoagulants could also be offered to more sub-groups, such as frail, elderly patients, and patients at high risk of recurring VTE events or high risk of bleeding complications, when considering the lower risk of bleeding complications.

In conclusion, we found a shift in treatment from of warfarin to a predominance of NOACs during the last decade. We found a slight increase in rate of PE cases in Region Stockholm. Further follow-up in relation to treatment complications, including bleeding events with different treatments, is warranted. Besides, further analyses of VTE events in relation to different cancers are warranted, especially on the time aspect, i.e., whether a VTE event could be the first sign of a cancer, or if VTE events occur later on after a cancer diagnosis.

## Electronic supplementary material

Below is the link to the electronic supplementary material.

Supplementary material 1 (DOCX 25 kb)
