# Supplementary material for: Venous thromboembolism 2011–2018 in Stockholm: a demographic study
Source: J Thromb Thrombolysis. 2019 Oct 1;48(4):668–73. doi: 10.1007/s11239-019-01966-y (PMC6800867; doi:10.1007/s11239-019-01966-y)
Supplement: Supplementary file 1 — Supplementary material 1 (DOCX 25 kb) [file 11239_2019_1966_MOESM1_ESM.docx]

**Supplementary Table 1 - Definitions of baseline co-morbidities by ICD-10, primary care codes and procedure codes.**

| **Diagnosis** | **ICD-code beginning with** |
| --- | --- |
| Alcohol abuse | E244, F10, G312, G621, G721, I426, K292, K70, K860, O354, P043, Q860, T51, Y90-91, Z502, Z714 |
| Anaemia | D50-64 |
| Any severe bleed | I60-62, I690-I692, S064-S066, I850, I983, K25-28 (sub codes 0-2 and 4-6 only), K625, K922, D500, D629, J942, I312, H431, H356 |
| Atrial fibrillation | I48 |
| Cancer | entire C-series |
| COPD/Emphysema | J43-44 |
| Dementia | F00-F03 |
| Diabetes | E10-E14 |
| Frequent falls (more than one registration) | W00-19 |
| Gastric duodenal bleeding | K25-28 (sub codes 0-2 and 4-6 only) |
| Heart failure | I50 |
| Hypertension | I10-I15 |
| Ischemic stroke, arterial embolism, and stroke, unspecified | I63, I64, I679, I693, I694, I698, I67-, I69-, Z866A, Z866B, Z867C, G450, G451, G452, G453, G458, G45.9, G45-, I74 |
| Intracranial bleeding | I60-I62, I690-I692, S064-S066 |
| Liver disease | K70-77 |
| Obesity | E65-66 |
| Renal disease | N17, N183, N184, N185, N189 |
| Vascular disease | I20-I25, I70, I739 |
| Venous thromboembolism | I26, I80 (I80.0 excluded), I82 (I82.1 excluded), I27.82 |

**Supplementary Table 2 – ATC-codes of the studied treatments**

| **Treatment** | **ATC-code beginning with** |
| --- | --- |
| Acetylsalicylic acid (aspirin) | B01AC06 |
| Antidepressants | N06A |
| Antihypertensive treatments | C03 C07 C08 C09 |
| Anticonception | G03A G03F |
| Anticonception2 | G03AA G03AB |
| Apixaban | B01AF02 |
| Clopidogrel | B01AC04 |
| Dabigatran | B01AE07 |
| Edoxaban | B01AF03 |
| Lipid lowering treatments | C10 |
| Low molecular weight heparin (LMWH) | B01AB04 B01AB05 B01AB10 |
| Insulin | A10A |
| Non-vitamin K oral anticoagulant (NOAC) | B01AE07 B01AF01 B01AF02 B01AX06 B01AF03 |
| Oral anticoagulant (OAC) | B01AE07 B01AF01 B01AF02 B01AX06 B01AF03 B01AA |
| Oral diabetes treatments | A10B |
| Prasugrel | B01AC22 |
| Rivaroxaban | B01AF01,B01AX06 |
| Ticagrelor | B01AC24 |
| Proton pump inhibitors | A02BC |
| Warfarin | B01AA |

Supplementary Table 3. New cases of venous thromboembolism in Region Stockholm 2011-2018, with the most common cancer diagnoses (with numbers, N, and proportion of cohort in percentage), grouped after frequency. The diagnoses might overlap.

| ICD-10 code | Cancer type | N | Proportion |
| --- | --- | --- | --- |
| C44 | Skin cancer | 721 | 19.5 |
| C61 | Prostate cancer | 680 | 18.4 |
| C50 | Breast cancer | 540 | 14.6 |
| C77 | Metastatic lymph node | 512 | 13.9 |
| C78 | Metastasis in lung, thorax, liver or  other gastrointestinal organs | 508 | 13.8 |
| C79 | Other sites of metastasis | 389 | 10.5 |
| C34 | Lung cancer | 320 | 8.7 |
| C18 | Colon cancer | 287 | 7.8 |
| C67 | Bladder cancer | 189 | 5.1 |
| C20 | Rectal cancer | 169 | 4.6 |

Supplementary Table 4. All patients with a recorded diagnosis of VTE in Region Stockholm who claimed either OAC or LMWH within 30 days before or after the index date the years 2011-2018 (data without age-standardization).

| Age group | 2011 | 2012 | 2013 | 2014 | 2015 | 2016 | 2017 | 2018 | All years |
| --- | --- | --- | --- | --- | --- | --- | --- | --- | --- |
| 0-19 years | 23 | 33 | 19 | 27 | 30 | 37 | 34 | 30 | 233 (0.8%) |
| 20-39 years | 307 | 314 | 335 | 329 | 354 | 352 | 383 | 326 | 2700 (9.8%) |
| 40-59 years | 855 | 823 | 810 | 827 | 887 | 906 | 853 | 880 | 6841 (24.7%) |
| 60-79 years | 1479 | 1466 | 1520 | 1516 | 1493 | 1639 | 1621 | 1727 | 12461 (45.1%) |
| 80-99 years | 658 | 672 | 653 | 659 | 685 | 685 | 694 | 703 | 5409 (19.6%) |
| ≥ 100 years | 2 | 1 | 1 | 2 | 1 | 2 | 1 | 4 | 14 (0.1%) |
| Totally | 3324 | 3309 | 3338 | 3360 | 3450 | 3621 | 3586 | 3670 | 27658 |

Supplementary Table 5. All patients with a recorded diagnosis of pulmonary embolism in Region Stockholm who claimed either OAC or LMWH within 30 days before or after the index date the years 2011-2018. (data without age-standardization).

| Age group | 2011 | 2012 | 2013 | 2014 | 2015 | 2016 | 2017 | 2018 | All years |
| --- | --- | --- | --- | --- | --- | --- | --- | --- | --- |
| 0-19 years | 5 | 8 | 7 | 5 | 13 | 7 | 11 | 6 | 62 (0.6%) |
| 20-39 years | 82 | 89 | 78 | 106 | 108 | 108 | 119 | 92 | 782 (7.2%) |
| 40-59 years | 269 | 233 | 277 | 277 | 294 | 286 | 266 | 279 | 2181 (20.1%) |
| 60-79 years | 586 | 605 | 646 | 664 | 629 | 714 | 762 | 772 | 5378 (49.5%) |
| 80-99 years | 297 | 292 | 306 | 301 | 296 | 305 | 345 | 326 | 2468 (22.7%) |
| ≥ 100 years | 2 | 0 | 0 | 0 | 0 | 0 | 0 | 0 | 2 (0.0%) |
| Totally | 1241 | 1227 | 1314 | 1353 | 1340 | 1420 | 1503 | 1475 | 10873 |

Supplementary Table 6. Population in Region Stockholm the years 2011-2018

| Age group | 2011 | 2012 | 2013 | 2014 | 2015 | 2016 | 2017 | 2018 |
| --- | --- | --- | --- | --- | --- | --- | --- | --- |
| 0-19 years | 497980 | 503966 | 509997 | 519247 | 525299 | 531870 | 541841 | 554951 |
| 20-39 years | 594205 | 606485 | 616669 | 633428 | 640510 | 640691 | 650864 | 669495 |
| 40-59 years | 540851 | 551609 | 561165 | 573871 | 585136 | 591866 | 599807 | 611154 |
| 60-79 years | 340017 | 345455 | 351251 | 358893 | 365129 | 370631 | 377041 | 385924 |
| 80-99 years | 85000 | 84325 | 83600 | 83433 | 82866 | 82898 | 83388 | 84730 |
| ≥ 100 years | 405 | 440 | 441 | 454 | 451 | 447 | 429 | 453 |
| Totally | 2058458 | 2092280 | 2123123 | 2169326 | 2199391 | 2218403 | 2253370 | 2306707 |
